# Functional traits variation explains the distribution of *Aextoxicon punctatum* (Aextoxicaceae) in pronounced moisture gradients within fog-dependent forest fragments

**DOI:** 10.3389/fpls.2015.00511

**Published:** 2015-07-23

**Authors:** Beatriz Salgado-Negret, Rafaella Canessa, Fernando Valladares, Juan J. Armesto, Fernanda Pérez

**Affiliations:** ^1^Instituto HumboldtBogotá, Colombia; ^2^Departamento de Ecología, Pontificia Universidad Católica de ChileSantiago, Chile; ^3^LINCGlobal, Museo Nacional de Ciencias Naturales, Consejo Superior de Investigaciones CientificasMadrid, Spain; ^4^Instituto de Ecología y BiodiversidadSantiago, Chile

**Keywords:** climate change, fog-dependent forest, fragmentation, hydraulic traits, intraspecific phenotypic variability, leaf traits, moisture gradient, phenotypic integration

## Abstract

Climate change and fragmentation are major threats to world forests. Understanding how functional traits related to drought tolerance change across small-scale, pronounced moisture gradients in fragmented forests is important to predict species’ responses to these threats. In the case of *Aextoxicon punctatum*, a dominant canopy tree in fog-dependent rain forest patches in semiarid Chile, we explored how the magnitude, variability and correlation patterns of leaf and xylem vessel traits and hydraulic conductivity varied across soil moisture (SM) gradients established within and among forest patches of different size, which are associated with differences in tree establishment and mortality patterns. Leaf traits varied across soil-moisture gradients produced by fog interception. Trees growing at drier leeward edges showed higher leaf mass per area, trichome and stomatal density than trees from the wetter core and windward zones. In contrast, xylem vessel traits (vessels diameter and density) did not vary producing loss of hydraulic conductivity at drier leeward edges. We also detected higher levels of phenotypic integration and variability at leeward edges. The ability of *A. punctatum* to modify leaf traits in response to differences in SM availability established over short distances (<500 m) facilitates its persistence in contrasting microhabitats within forest patches. However, xylem anatomy showed limited plasticity, which increases cavitation risk at leeward edges. Greater patch fragmentation, together with fluctuations in irradiance and SM in small patches, could result in higher risk of drought-related tree mortality, with profound impacts on hydrological balances at the ecosystem scale.

## Introduction

Reductions in precipitation expected under climate change and increasing forest fragmentation are major threats to temperate forests worldwide (Breshears et al., 2005; Echeverría et al., 2006; Choat et al., 2012). Particularly sensitive are forests located in the boundary with drier formations, where drought intensification may be fundamentally important for the persistence of forest communities (Pockman and Sperry, 2000; Engelbrecht et al., 2007; Choat et al., 2012; Salgado-Negret et al., 2013). As a consequence, improved understanding of functional trait variation in relation to drought tolerance becomes critical for modeling and predicting tree species responses to future climate change (Anderegg et al., 2013).

Variation in functional traits can derive from phenotypic plasticity, genetic variation, developmental instability, and direct effects of stress on plant performance, or a combination of these mechanisms (Valladares et al., 2007; Matesanz et al., 2010; Gianoli and Valladares, 2012). In recent years, interest in drought-resistance trait variation at the intraspecific level has increased (Choat et al., 2007; Cornwell and Ackerly, 2009; Figueroa et al., 2010; Fajardo and Piper, 2011), because of its relevance to understanding plant species responses to drought stress and the maintenance of biodiversity (Violle et al., 2012). Studies have often focused on species distributions across broad geographic ranges and stress conditions (Choat et al., 2007; Figueroa et al., 2010; Fajardo and Piper, 2011; Vasey et al., 2012; Jacobsen and Pratt, 2013). However, intraspecific variation of functional plant traits across pronounced environmental gradients at small spatial scales can also provide clues to identifying species responses to key environmental factors, such as water availability, and their interactions with wide spread global change threats such as fragmentation (Matesanz et al., 2009).

In semiarid regions, ecosystems that depend on coastal fogs for water supply (del-Val et al., 2006; Hildebrandt and Eltahir, 2006; Katata et al., 2010; Vasey et al., 2012) represent an interesting case with respect to acute moisture gradients. In such ecosystems, fog interception by vegetation is the primary or even the only source of moisture during prolonged dry periods (Dawson, 1998; del-Val et al., 2006; Ewing et al., 2009). Fog influx creates pronounced asymmetries between windward to leeward edges of forest patches (Weathers et al., 2000; del-Val et al., 2006; Ewing et al., 2009; Stanton et al., 2013), as well as among different-size patches with contrasting edge effects. Fragmentation enhances sensitivity to current and future changes in fog water supply (Hildebrandt and Eltahir, 2006; Gutiérrez et al., 2008; Johnstone and Dawson, 2010). Changes in fog frequency and intensity are predicted to occur in these areas due to changes in sea-surface temperature and the height of the temperature inversion layer (Cereceda et al., 2002; Garreaud et al., 2008), together with changes in other forest features affecting fog capture (Hildebrandt and Eltahir, 2006).

An emblematic example of fog-dependent forests found in semiarid Chile (30°S) is the northernmost extension of temperate rainforest on coastal hilltops of the semiarid region. Here, a mosaic of rain forest patches of different sizes occurs immersed in a xerophytic shrub land matrix (Barbosa et al., 2010). The dominant tree species in all forest patches is the southern South American endemic *Aextoxicon punctatum* Ruiz and Pav, belonging to the monotypic and isolated family Aextoxicaceae. This species is broadly distributed in temperate rain forests of western South America. In Fray Jorge, it occurs in forest patches of all sizes and throughout the soil moisture (SM) gradient produced by fog influx from windward to leeward edges (del-Val et al., 2006). Moreover, population genetic studies suggest that gene flow via seed dispersal across neighboring patches in this patchy landscape has been highly significant (*F*st < 0.05) during recent history (Nuñez-Ávila et al., 2013). Patterns of tree radial growth and regeneration dynamics of *A. punctatum* in this forest have shown constant growth and continuous regeneration for 200 years, despite a declining trend in rainfall during the last century. This suggests that this species can survive extreme temporal fluctuations in water availability (Gutiérrez et al., 2008). Understanding the ability of *A. punctatum* to withstand spatial and temporal fluctuations in water availability requires improved knowledge of the mechanisms involved in drought tolerance and vulnerability to the combined effects of increased water shortage and forest fragmentation.

Plants often respond to water deficit by modifying leaf traits and decreasing transpirational water losses through reductions in stomatal size and density, greater trichome density (TM; Fahn, 1986; Baldini et al., 1997), and enhanced leaf mass per unit area (LMA; Niinemets, 2001; Poorter et al., 2009). Large number of short and narrow vessels per unit area are also adaptive under arid conditions and reduces the chances of hydraulic embolism (Pockman and Sperry, 2000; Carlquist, 2001; Hacke et al., 2001; Jacobsen et al., 2007; Markesteijn et al., 2011a,b). The above-cited studies have generally focused on changes in mean trait values, while changes in trait variability [measured by the coefficient of variation (CV)] have received less attention (Violle et al., 2012). Likewise, comparative studies across moisture gradients have often ignored the phenotypic or morphological integration (Nicotra et al., 2007), which refers to coordinated variation of morphological traits (Cheverud et al., 1989; Murren, 2002; Pigliucci, 2003; Pigliucci and Preston, 2004) and it can result from different processes (Klingenberg, 2014) including pleiotropy or coordinated gene expression, functional relation among traits or development factors. Trait correlations among individuals within a population or species are generally used to characterize integration patterns (static integration sensu Klingenberg, 2014), but variation among related species or across ontogenetic stages has also been considered. Correlations between leaf (Wright et al., 2004) and hydraulic traits (Chave et al., 2009; Zanne et al., 2010) have been documented by several recent studies (Brodribb and Feild, 2000; Brodribb et al., 2002; Santiago et al., 2004; Wright et al., 2006; Meinzer et al., 2008; Baraloto et al., 2010). However, we lack information about how the environment can alter patterns of phenotypic integration (Nicotra et al., 1997, 2007; Wright et al., 2006). Nevertheless, studies of other groups of traits indicate that phenotypic integration should increase with environmental stress (Schlichting, 1989; Gianoli, 2004; Godoy et al., 2012).

This study explores the magnitude, variability and correlation patterns of leaf traits, xylem vessel traits and hydraulic conductivity of the rain forest tree *A. punctatum* across contrasting SM conditions, which occur within fog-dependent forest patches in semiarid Chile. A striking asymmetric pattern in these patches is that tree mortality increases toward the leeward edge and regeneration is enhanced toward windward edges (del-Val et al., 2006). Specifically, we addressed the following two hypotheses: (1) individuals that occur in drier leeward edges of forest patches may display traits that favor water conservation (lower stomatal and higher TD, and LMA) and minimize cavitation risk (lower vessel diameter and higher vessel density, and decreased hydraulic conductivity); (2) phenotypic variation and integration may increase in leeward edges due to greater environmental variability and increased water shortage.

## Materials and Methods

### Study Site and Species

**FIGURE 1:**
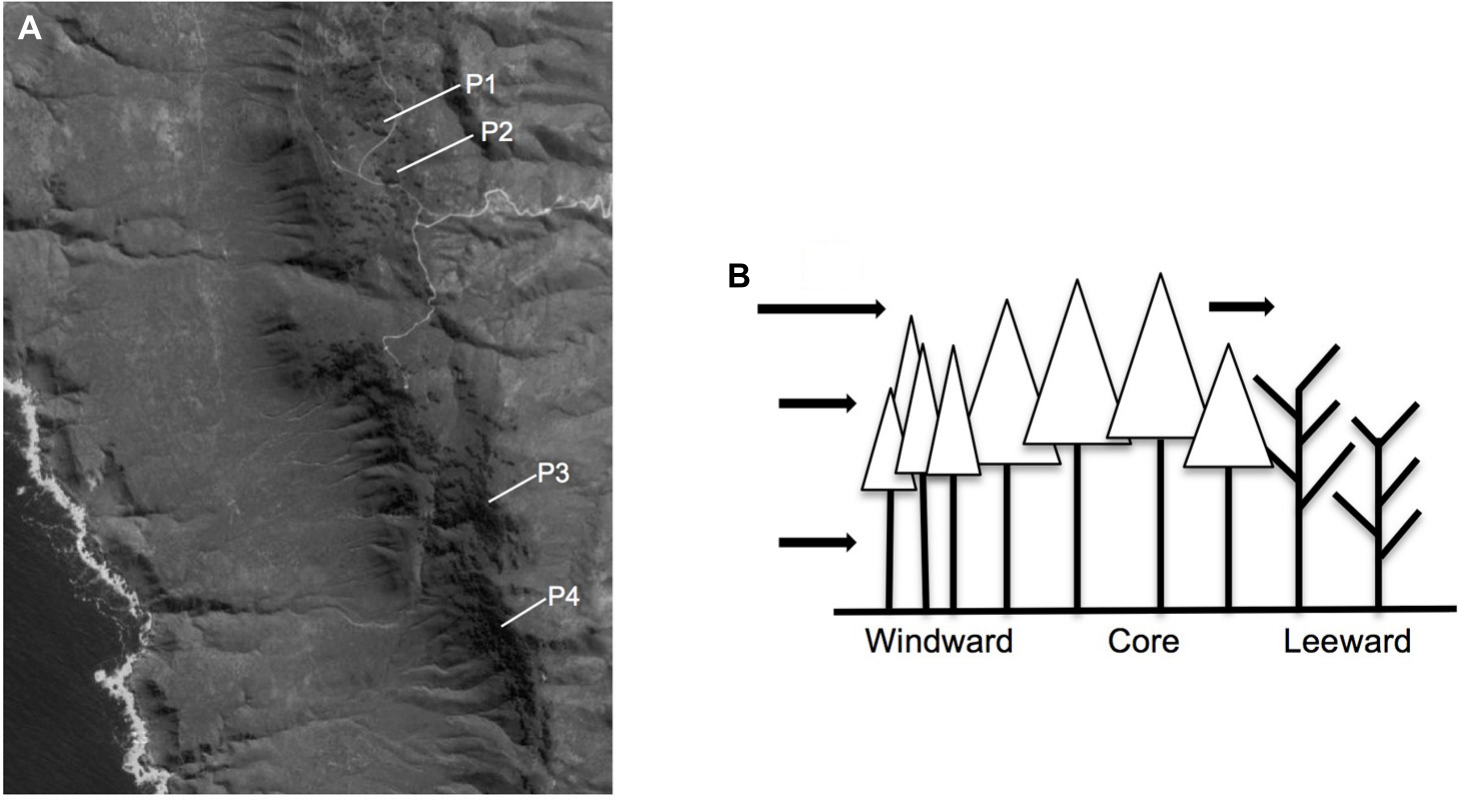
**(A)** Location of rain forest patches in Fray Jorge National Park, Chile, at 30°S. **(B)** Directionality of fog and atmospheric resource inputs to forest patches in Fray Jorge.

Fray Jorge National Park (30°40′S 71°30′W) comprises the northernmost patches of Chilean temperate rainforests under the direct influence of maritime fog. A mosaic of about 180 forest patches ranging in size from 0.1 to 36 ha are spread out on the summits of coastal mountains at an elevation of 450–660 m (**Figure 1**; Barbosa et al., 2010). Forest patches are surrounded by a matrix of semiarid shrub vegetation, in correspondence with the Mediterranean-arid regional climate, with a mean annual rainfall of 147 mm concentrated during the winter months (May to August) and a mean annual temperature of 13.6°C (López-Cortés and López, 2004). Fog is the major water input above 400 m elevation, especially during spring and summer months, such that fragments receive at least an additional 200–400 mm of water annually via throughfall and stem flow (del-Val et al., 2006). In these fog-dependent forests, SM is spatially heterogeneous due to fog interception by trees, creating an asymmetric SM distribution from windward to leeward edges (Stanton et al., 2013). This within-patch environmental gradient has important effects on the dynamics of tree species, yielding an asymmetric distribution of tree regeneration and mortality from windward to leeward edge of patches (del-Val et al., 2006; Gutiérrez et al., 2008). Those patches are dominated by *A. punctatum*, a tree species with sclerophyllous leaves and traits related to drought tolerance such as small vessels, and low hydraulic conductivity and potential at turgor loss point (Salgado-Negret et al., 2013). Wood has inconspicuous growth rings (with fewer and narrower vessels in late wood) and conserves several primitive features, such as the presence of tracheids and scalariform perforation plates with extensive pit-membrane remnants (Carlquist, 2003).

### Sampling Design and Soil Moisture

To assess intraspecific variation in leaf traits, xylem vessel traits and hydraulic conductivity of *A. punctatum* across the SM gradient produced within patches by fog influx, we sampled four forest patches separated by at least 200 m from one another. For logistic reasons, due to the number of simultaneous measurements per patch, we selected two small (<1 ha) and two large patches (>20 ha) corresponding to the extremes of the distribution of patch sizes in the mosaic studied (**Table 1**; Barbosa et al., 2010). Patches were subdivided into three zones according to spatial variation in fog influx: windward edge, patch core, and leeward edge, and five individuals of *A. punctatum* (dbh > 10 cm) per zone per patch were sampled (5 trees × 3 zones × 4 forest patches = 60). Five measurements of volumetric SM were recorded during summer (January 2010) for each tree using a hand-held TDR probe (Fieldscout TDR 100, Spectrum Technologies, Illinois, USA). Measurements were collected after clearing away leaf litter and subaerial roots directly beneath the tree crown. To assess the real water status of plants, we measured leaf water potentials at predawn (ψ_PD_, MPa) between 0500 and 0700 h and at midday (ψ_MD_, MPa) between 1100 and 1300 h during summer using a pressure chamber (Scholander-type, model 1000 PMS, Albany, NY, USA). We measured five leaves of each of the five individuals per zone.

**Table 1:** Characterization of forest patches, including differences in mean values for microclimatic variables and relative basal area for all live stems (>5 cm dbh; Gutiérrez et al., 2008; Barbosa et al., 2010).

	P1	P2	P3	P4
Patch area (ha)	0.21	0.28	36.08	23.76
Altitude (m)	529	566	635	639
Slope (%)	1	11	42	38
Throughfall (mm)	31.10 ± 21.31	49.91 ± 43.16	29.56 ± 18.05	37.38 ± 22.55
Stemflow (mm)	0.10 ± 0.06	0.25 ± 0.06	0.69 ± 1.07	1.00 ± 0.99
Mean temperature (°C)	11.8 ± 1.6	11.46 ± 1.75	11.29 ± 1.51	10.95 ± 1.42
Mean relative humidity (%)	91.33 ± 4.00	94.98 ± 3.04	95.96 ± 3.73	95.12 ± 4.63
Tree basal area (m^2^ ha^-1^)	61.64	49.41	125.12	102.61
Basal area *Aextoxicon punctatum* (%)	49	75.7	46.4	88.8

## Leaf Traits

Ten mature, fully expanded leaves without herbivore damage were taken from each of five sample trees per patch zone. Leaves were scanned (EPSON Stylus TX200) and analyzed using ImageJ software (http://imagej.nih.gov/ij/) to determine leaf area (LA), and then dried for 48 h at 65°C to obtain leaf dry mass (g) and then calculate leaf mass per area (LMA) in g m^-2^ (Cornelissen et al., 2003). One leaf per individual was prepared to determinate TD and stomatal density (SD). Leaves were kept in Jeffrey solution (chromic acid at 10% and nitric acid at 10% in equal parts) for 48 h, until the epidermis could be easily separated from the mesophyll. Later, the epidermis was dyed in diluted methylene blue and stomatal and trichome densities were measured on one spot of 1 mm diameter located halfway along the length of the leaf using ImageJ software (http://imagej.nih.gov/ij/).

### Hydraulic Conductivity

We collected a sample of branches (10–15 mm in diameter) during January 2011 (austral summer). Samples were taken from the outer crown of the same set of trees sampled for leaf traits measurements with the purpose of assessing hydraulic conductivity, i.e., water flux through a unit length of stem divided by the pressure gradient (*K*_h_, in kg m^-1^ s^-1^ MPa^-1^), following Sperry et al. (1998). Branches were cutoff in the morning (between 6:00 and 9:00 am) when water demands and xylem tension had the lowest values for the day. Immediately after first cutting, branches were re-cut under water, eliminating a segment of about 20 cm long. This segment was longer than the maximum vessel length in this species, previously determined in a subset of 10 individuals by injecting air with a hand pump on a 1.5 m long segment and cutting it back distally until the first bubbles were seen (Ewers and Fisher, 1989). Branches were subsequently transported to the field station inside dark bags containing a moist paper towel to prevent desiccation. Within 5 h after cutting, native hydraulic conductivity was measured at the field station (located 45 min from sampling sites). Distal ends of each branch were trimmed at 30 cm under water with a razor blade to clear any artificially blocked vessel. The branch segment measured was two times as long as the maximum vessel length, and we therefore assumed that all conduits were closed, thus avoiding overestimation of hydraulic conductivity (Chiu and Ewers, 1993). While submerged, the basal end of the branch was connected to a fluid column fed by a reservoir of 10 mM KCl solution elevated to a height of 1 m (providing a constant pressure of 9.8 kPa), while the apex end of the branch was wrapped with parafilm. An electronic balance recorded KCl solution flux as increase in sample mass every 15 s. Measurements were made when an approximately constant flow was observed for at least 3 min. Afterward, a subset of branches was flushed with KCl solution at a pressure of 170 kPa for 10–15 min to remove embolism (Sperry et al., 1987) and hydraulic conductivity was measured again at its maximum capacity (*K*_MAX_). To standardize the flow of water per unit sapwood area and obtain sapwood specific hydraulic conductivity (*K*_s_ native, kg MPa^-1^ m^-1^ s^-1^), we divided K_h_ by the cross-sectional area of the conductive xylem (see hydraulic anatomy below). Thus, hydraulic conductivity was made comparable among segments of different diameters. *K*_s_ native was compared with sapwood specific hydraulic conductivity at maximum capacity to obtain the percentage of loss conductivity (PLC), estimated as (*K*_MAX_ – *K*_s_ native)/*K*_MAX_. These data were available for a subset of three individuals per zone in only two patches.

### Xylem Vessel Traits

To visualize the conductive wood area, the same stems were perfused with safranin dye using positive pressure by syringe connected to the cut end of the branch to introduce the dye into stems. A cross-sectional area of the upper distal end of the stem was photographed with a digital camera mounted on a microscope, at 10x and the image processed using the imaging software SigmaScan Pro 5 (SPSS Inc.) to determine vessel diameter (VDi) and density (VD). The mean values and the 75th and 90th percentiles of vessel diameter were recorded to assess differences across forest patch zones.

### Data Analysis

Differences across forest patch zones (windward and leeward edges and core) in leaf traits, xylem vessel traits and hydraulic conductivity were explored using principal component analysis. Linear models, fitted by generalized least squares (GLSs) with a restricted maximum log-likelihood, were also applied to assess differences among zones for the variables SD, LMA, VDI (mean, 75th and 95th percentile) and VD. Analyses were conducted independently for each variable using R version 3.1.1 (R Development Core Team, 2014; Pinheiro et al., 2015; R-package nlme module). The models contained three levels for the factor Zone nested within Forest Patch (Four levels: P1, P2, P3, and P4), as fixed effect [Z (P)]. Shapiro–Wilk and Levene’s tests were performed to check for normality and homoscedasticity of residuals. Given that SD showed heteroscedasticity, a constant variance structure was added using the function varIdent in R package nlme (Pinheiro et al., 2015). In this way, the variance was independently specified for each zone and fragment. Variation in TD and SM (which did not have normally distributed residuals) were analyzed using generalized linear models (GLMs) with a Poisson error structure for the first variable, and a Gamma error structure for the second one. All analyses were conducted in R version 3.1.1 (R Development Core Team, 2014) again with Zone nested within Forest Patch as fixed factor. Significance of the Z (P) term was tested with a *F*-test for traits with normally distributed errors and with a Chi-test for traits with non-normally distributed errors. Multiple comparison tests for differences among zones within each forest fragment (12 comparisons per trait) were performed using the function glth in R-package multcomp (Hothorn et al., 2008) and the default single-step method to adjust P-values for multiple tests.

Given that we did not find clear differences in mean values among patch zones in small forest patches (P1 and P2), we examined shifts in the spread and phenotypic integration among zones only in the large patches. Because the two large patches studied showed similar patterns in mean values, we pooled these data for further analyses (10 individuals per zone). The variability between zones was compared using the CV (CV = SD/mean), which is appropriate to compare variability of groups with different means. Pairwise comparisons between zones were conducted using the non-parametric bootstrap test proposed by Cabras et al. (2006), recommended for small sample sizes (*n* ≥ 10) and non-normally distributed data. Cabras et al. (2006) proposed the statistic *Z*_D_ = *T*_D_/√*V*_D_; where *T*_D_ is the absolute difference between the CV of two populations and *V*_D_ is the variance of *T*_D_ estimated by bootstrap resampling. The *p*-value was estimated by bootstraping as: #{*Z*_Db_^∗^ ≥*Z*_D_ }/B+1; where *Z*_Db_^∗^ is the value of the statistic *Z*_D_ for *b* = 1,2,…., B bootstrap replications. *Z*_D_ distributions and *p*-values were estimated with 10,000 iterations. The analyses resulted in three comparisons for each trait, and therefore Bonferroni correction for multiple comparisons was performed (significance at 0.05/3 = 0.017). All data analyses were performed in R version 3.1.1 (R Development Core Team, 2014).

To assess phenotypic integration, we constructed 5*5 correlation matrices with morphological traits for each zone (MCs) and for all individuals using Pearson’s correlation coefficients to test the relationships for every pair of traits. The magnitude of character integration (INT) for each zone and for large patch data was estimated from the variance of eigenvalues of each correlation matrix (Wagner, 1984; Cheverud et al., 1989). A 95% confidence interval of INT was estimated by bootstrapping the original log-transformed data.

## Results

### Within-Patch Moisture Gradient and Leaf Water Potential

In all patches volumetric SM varied substantially among zones, with leeward edges significantly drier than the other two microhabitats (**Table 2**). Differences in SM among patch zones were reflected in lower ψ_PD_ and ψ_MD_ at leeward edges.

**Table 2:** Differences in soil moisture (SM), leaf water potential, leaf traits, xylem vessel traits and hydraulic conductivity among individuals of *A. punctatum* growing in different zones of small (P1, P2) and large forest patches (P3, P4) in Fray Jorge.

	Soil	-ψ_PD_	-ψ_MD_	SD	TD	LMA	VD	VDi			K_s_
								75th	90th	Mean	
**P1**				
W	7.9^a^	0.27^a^	1.00^a^	133^a^	8.0^a^	193^a^	336^ab^	19.2	21.4	16.8^a^	0.35^a^
C	10.2^a^	0.33^a^	1.27^a^	125^a^	6.0^a^	161^a^	366^a^	18.8	21.3	16.6^a^	0.22^a^
L	4.8^b^	0.60^b^	1.67^b^	141^a^	9.8^a^	190^a^	272^b^	20.3	22.9	18.0^a^	0.16^a^
**P2**				
W	13.0^a′^	0.82^a′^	1.53^a′^	154^a′^	10.6^a′^	214^a′^	325^a′^	18.7	21.0	16.5^a′^	0.43^a′^
C	14.0^a′^	0.80^a′^	1.56^a′^	135^a′^	7.0^a′^	186^a′^	295^a′^	18.9	21.1	16.7^a′^	0.26^a′b′^
L	5.2^b′^	0.89^a′^	1.98^b′^	153^a′^	9.2^a′^	231^a′^	309^a′^	19.7	22.1	17.3^a′^	0.11^b′^
**P3**				
W	8.4^a′′^	0.24^a′′^	0.69^a′′^	108^a′′^	4.6^a′′^	122^a′′^	293^a′′^	21.7	23.8	18.6^a′′^	0.40^a′′^
C	14.8^b′′^	0.14^a′′^	0.70^a′′^	135^b′′^	5.2^a′′^	98^a′′^	304^a′′^	19.2	21.1	16.7^b′′^	0.39^a′′^
L	5.0^c′′^	0.77^b′′^	1.39^b′′^	200^c′′^	13.2^b′′^	210^b′′^	328^a′′^	18.5	20.9	16.3^b′′^	0.13^b′′^
**P4**				
W	10.1^a′′′^	0.05^a′′′^	0.76^a′′′^	108^a′′′′^	4.8^a′′′^	179^a′′′^	277^a′′′^	19.8	21.5	17.3^a′′′^	0.42^a′′′^
C	14.4^a′′′^	0.06^a′′′^	0.72^a′′′^	111^a′′′^	4.8^a′′′^	180^a′′′^	300^a′′′^	19	20.9	16.7^a′′′^	0.34^a′′′^
L	4.4^b′′′^	0.88^b′′′^	2.03^b′′′^	141^b′′′^	8.2^b′′′^	336^b′′′^	291^a′′′^	20.2	22.6	17.5^a′′′^	0.07^b′′′^
**Zone (patch)**				
p	*D*_8_	*F*_4,48_	*F*_4,48_	*F*_4,48_	D_8_	*F*_4,48_	*F*_4,48_	*F*_4,48_	*F*_4,48_	*F*_4,48_	*F*_4,48_
	11.9	19.9	25.9	17.3	42.6	21.7	2.3	1.93	1.79	2.3	27.2
	< 0.001	<0.001	<0.001	<0.001	<0.001	<0.001	0.04	0.07	0.10	0.04	<0.001

*Traits are abbreviated as: Soil, SM (%); -ψ_PD_, leaf water potential at predawn (MPa); -ψ_MD_, leaf water potential at midday (MPa); SD, stomatal density (N/mm^2^); TD, trichome density (N/mm^2^); LMA, leaf mass area (g/m^2^); VD, vessel density (N/mm^2^); VDi, Vessel diameter (μm; mean, 75th and 90th percentiles); K_s_, native sapwood-specific hydraulic conductivity (kg MPa^-1^ m^-1^ s^-1^). *F*-values and deviances are shown for traits with normally and non-normally distributed errors, respectively. Different lower case letters represent statistically significant differences between zones for each trait and fragment at 5%. Values indicate the mean from 5 individuals per patch-zone.*

### Shifts in Mean Trait Values Across Zones within Patches

**FIGURE 2:**
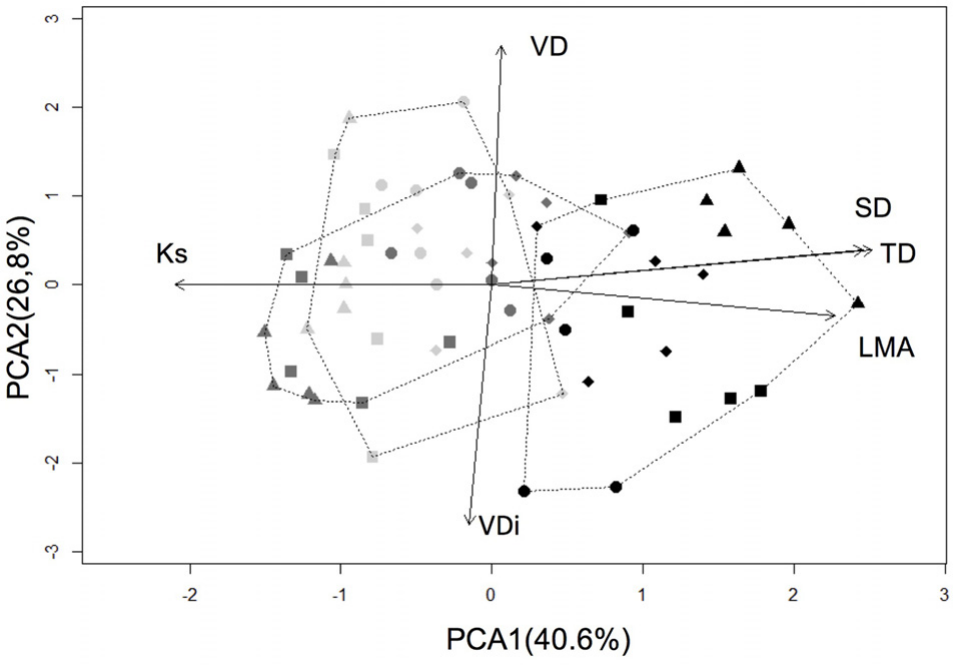
**Principal component analysis (PCA) of hydraulic and leaf trait variation for *Aextoxicon punctatum* trees among forest patches (small patches: P1, circle; P2, rhombus; large patches: P3, triangle; P4, square) and zones within patches (windward edge, dark gray; patch core, light gray; leeward edge, black) in Fray Jorge.** Minimum convex polygons for each zone are shown. See trait abbreviations in **Table 2**.

Plant traits also differed among patch zones as revealed by PCA. The first PCA axis, which explained 41% of trait variation, clearly separated leeward edge from other two wetter zones (**Figure 2**). This axis was positively correlated with traits related to water conservation strategy (TD and SD and LMA) and negatively correlated with *K*_s_ native. Then, higher values along the first PCA axis reflected stronger ability to conserve water and tolerate drought, but decreased water transport efficiency. The second PCA component explained an additional 27% of the total variance and it was dominated by the tradeoff between vessels diameter and density. However, it did not separate trees in different patch zones. Similar results were detected when each trait was analyzed separately using GLS or GLM. These analyses also provided evidence that trait variation was more pronounced in the large (P3 and P4) than in small forest fragments (P1 and P2). In large patches, trees growing at leeward edge showed higher SD, TD and LMA than in the wetter windward and core zones (**Table 2**). In contrast, vessel diameter and density were conserved among zones. No significant differences were observed in 75th and 90th percentiles of vessel diameter, and only P5 showed differences in the mean vessel diameter and only P1 in vessel density. Trees growing at leeward edge showed lower *K*_s_ native in fragments P2, P3, and P4 (**Table 2**). To assess whether reduction in *K*_s_ native at leeward edges reflected higher levels of embolism, we estimated *K*_MAX_ at maximum capacity and the percentage of loss conductivity (PLC) in three to five individuals per zone for two patches (P2 and P3). As expected, we found higher PLC values for trees in leeward edges of both fragments (P2: *F* = 10.52, *p* = 0.01; P5: *F* = 32.63, *p* < 0.001).

### Shifts in the Spread of Trait Values Across Zones within Patches

Given that we did not find clear differences in mean values among patch zones in small forest patches (P1 and P2), we examined shifts in the spread (CV) and phenotypic integration among zones only in the large patches. Two of the six traits evaluated showed significant trends in relation to forest patch zones (**Table 3**). CV of SD and hydraulic conductance (*K*_s_ native) were significant higher in the leeward edge than in the wetter core and windward zones (**Table 3**).

**Table 3:** Coefficient of variation of leaf traits, xylem vessel traits and hydraulic conductivity of *A. punctatum* trees among zones within large forest patches in semiarid Chile.

Traits	Windward	Core	Leeward
SD	0.05^a^	0.11^b^	0.21^c^
TD	0.20^a^	0.16^a^	0.29^a^
LMA	0.30^a^	0.34^a^	0.27^a^
VD	0.12^a^	0.12^a^	0.12^a^
VDi	0.06^a^	0.09^a^	0.07^a^
K_s_	0.28^a^	0.27^a^	0.72^b^

*Letters represent statistically significant differences between zones for each trait and fragment based on non-parametric bootstrap test (Cabras et al., 2006). Significance at 5% after Bonferroni correction for multiple comparisons (three per trait).*

### Shifts in Trait Correlations and the Extent of Phenotypic Integration within Patches

Phenotypic correlation matrices varied among zones within forest patches (**Table 4**). The most divergent matrix was that of the windward zone, showing similarity indices of -0.12 and 0.11 with respect to the leeward and core matrices. Phenotypic matrices of these last two zones (leeward and core) were more similar (similarity index = 0.71, *p* = 0.02), but often correlation coefficients were stronger in the drier leeward zone. Whereas mean *r*^2^ value for characters of trees in leeward areas was 0.40, this parameter was only 0.14 and 0.18 for trees in windward and core zones respectively. Integration values were also higher in the drier leeward zone (INT = 1.9, 95%CI: 1.32–3.39) than in windward (INT = 0.64, 95%CI 0.52–1.99) or core (INT = 0.92, 95%CI: 0.90–2.30) zones, but differences were not statically significant.

**Table 4:** Pearson correlation coefficients between leaf and hydraulic traits for trees from three zones that differ in SM, within four forest patches in Fray Jorge.

Zone	Traits	TD	LMA	VD	VDi
Windward	SD	0.55 (0.09)	-0.03 (0.93)	-0.56 (0.09)	0.16 (0.65)
	TD		-0.22 (0.54)	-**0.76 (0.0009)**	0.11 (0.74)
	LMA			0.00 (0.99)	-0.27 (0.44)
	VD				-0.16 (0.65)
Core	SD	0.34 (0.34)	-**0.87 (0.001)**	0.04 (0.91)	0.01 (0.96)
	TD		-0.25 (0.48)	0.04 (0.90)	0.05 (0.88)
	LMA			-0.18 (0.61)	0.06 (0.85)
	VD				-**0.9 (0.0004)**
Leeward	SD	**0.74 (0.01)**	-**0.75 (0.01)**	0.32 (0.37)	-0.29 (0.40)
	TD		-**0.76 (0.01)**	0.41 (0.23)	-0.46 (0.17)
	LMA			-**0.75 (0.01)**	**0.63 (0.05)**
	VD				-**0.73 (0.01)**

*p-values are shown in parentheses. Significant correlations among traits (*p* < 0.05) are indicated in bold.*

## Discussion

The temperate rainforest tree *A. punctatum* showed considerable variation in leaf traits across soil-moisture gradients produced by fog interception by the tree canopy. Notably, leaf trait variation occurred within structurally asymmetric forest fragments (del-Val et al., 2006; Stanton et al., 2013) at spatial scales of 100 m or less. In contrast to foliar traits, those related to xylem anatomy of *A. punctatum* (vessel diameter and density) did not vary significantly within forest fragments in Fray Jorge, and therefore they were decoupled from the observed variation in leaf traits. Other hydraulic traits such as cavitation resistance have been described as conservative within species (Lamy et al., 2011) and within genera (Jacobsen and Pratt, 2013), which has been interpreted as the result of selection on adult trees to survive episodic drought (Pockman and Sperry, 2000).

The absence of variability in xylem anatomy of *Aextoxicon* trees in Fray Jorge forest patches was associated with lower hydraulic conductivity measured in the drier leeward edge, as *K*_s_ native was four times lower for trees in the core or windward zones of patches. Reduced conductivity at the leeward edge might be explained by higher levels of native embolism, because PLC values at leeward edges were five times higher than PLC values measured in trees located in the core zones of patches P2 (small) and P3 (large). However, it is possible that differences between zones in native *K*_s_ might be due to different levels of embolism induced artificially. Wheeler et al. (2013) showed that sampling methods could induce a degree of embolism, which is a function of xylem tension. In the case of *A. punctatum*, artificial embolism is unlikely because this tree species has scalariform perforation plates (Nishida et al., 2006), and according to Wheeler et al. (2013), this type of plates are able to trap any entering bubbles right below to the entry-point, and therefore, bubbles should be removed when stems are cut off prior to *K*_s_ measurements. Additionally, *K*_s_ native was measured in the mornings when water demands and xylem tension had the lowest values for the day.

To determine whether leaf trait differentiation among patch zones is due to plastic responses or to local adaptation, it is necessary to compare among trees grown in common gardens or reciprocal transplant experiments. However, indirect evidence based on distances between patches and dispersal distances of *Aextoxicon* seeds dispersed by birds suggest that gene flow should occur among zones within forest patches as well as among patches (Nuñez-Ávila et al., 2013), and hence differences among trees in different patch zones are likely due to plasticity. Thus, leaf phenotypic plasticity in response to within patch differences in water availability is likely involved in the persistence of this tree species across a range of habitats.

Trees with higher LMA, trichome and stomatal densities grew more often in leeward edges, where water availability was two to three times lower than in the patch core zone and windward edges of patches. These differences in leaf traits can be related to water conservation strategies (Chapin, 1980). Leaves that are more dense and rigid (higher LMA) have smaller transpiring surfaces, hence reducing wilting and water requirements (Poorter et al., 2009). Greater leaf pubescence increases boundary layer resistance, decreases transpirational water losses (Fahn, 1986; Baldini et al., 1997), and also enlarges the surface available for water uptake by leaves (Grammatikopoulos and Manetas, 1994; Savé et al., 2000). The observed increment in leaf SD in trees growing at the drier leeward edge of patches is less intuitive, because greater stomatal densities are often associated with higher transpiration and water loss. However, stomata in *A. punctatum* leaves are sunken and located in the abaxial epidermis. Sunken stomata generally reduce leaf transpiration (Jordan et al., 2008) and facilitate CO_2_ diffusion in thick, hard leaves (Hassiotou et al., 2009). High stomatal densities in the drier leeward edge may probably compensate for the greater internal resistance to CO_2_ uptake by thicker and denser leaves (with higher LMA). In addition, long-lived leaves with higher LMA can exhibit higher stomatal densities as a ‘backup’ mechanism, in case that some stomata become inactive, i.e., dust blocked (Hassiotou et al., 2009).

Two of the four traits that showed differences in mean values across zones within fragments, also showed differences in their degree of variability. For stomatal densities and K_s_ native, the CV was greater for trees in the drier leeward edge. This patch zone is not only drier but also subjected to higher fluctuations in irradiance and temperature and therefore SM compared to core and windward zones of patches. These results agree with other studies showing increasing number of alternative phenotypes with increasing resource heterogeneity (Sultan, 1987; Lortie and Aarssen, 1996; Balaguer et al., 2001). In the case of *A. punctatum*, the higher CV for SD of trees in leeward habitats may be related to successive generations of leaves experiencing contrasting environments and therefore promoting alternative phenotypes. In contrast, the uniformity of xylem vessel traits may indicate high environmental canalization, due to the strong connection of hydraulic properties with water transport and survival, which enables organisms to maintain the highest possible level of fitness across environments (Debat and David, 2001). High canalization of hydraulic anatomy across all within-patch zones could lead to high K_s_ native variability at leeward edges.

We also found stronger correlations among leaf traits and greater level of phenotypic integration at decreasing levels of soil water availability within patches. Other studies of phenotypic integration also showed greater correlation values in heterogeneous environments (Schlichting, 1989; Nicotra et al., 1997; Gianoli, 2004), but the functional benefits or constraints on this pattern for plants have not been clearly established (Gianoli, 2004; Matesanz et al., 2010). Notably, we found that in the case of *A. punctatum* leaf traits varied rather independently of hydraulic traits, except for trees in the leeward edge, where LMA, vessel density and vessel diameter were correlated. In this heterogeneous and variable environment, which characterizes fragmented forests, functional coordination between stem conductive capacity and leaf hydraulic properties might be essential. Our results on this point contrast with other studies reporting coordinated variation of leaf and stem traits in forest trees (Brodribb and Feild, 2000; Brodribb et al., 2002; Santiago et al., 2004; Wright et al., 2006; Meinzer et al., 2008; but see Baraloto et al., 2010), and highlight the need to examine patterns of phenotypic integration across different environmental gradients.

Overall, this study demonstrates that *A. punctatum* leaf traits, but not xylem anatomy, vary within forest patches under contrasting soil-moisture conditions produced by fog interception patterns. The absence of similar plasticity in xylem traits of trees was correlated with a reduction in hydraulic conductance (K_s_ native and K_MAX_) at the drier leeward edge and it evidenced higher drought stress expressed by more negative ψ_PD_ in the leeward edges with respect to other zones. Although vessel diameters recorded for *A. punctatum* stems are in the smaller range of those reported for tree species in the literature (Ewers and Fisher, 1989; Chave et al., 2009; Zanne et al., 2010), high values of PLC recorded at leeward edge of patches revealed that soil water availability at this edge is insufficient to maintain a constant flux along stems. Indeed, we previously reported that hydraulic potential at midday (ψ_MD_) for individuals of *A. punctatum* growing at leeward edges frequently fell below the turgor loss point (π_tlp_), suggesting intense drought stress (Salgado-Negret et al., 2013).

Recent climate change scenarios for Chile (CONAMA, 2006) predict enhanced interannual variability in rainfall, greater intervals between extremely wet and dry years, and particularly a decline in winter rainfall (concentrating >80% of annual rainfall) in the study area. However, rain contributes only a fraction (about 50% during low rainfall years) of the annual water budget in Fray Jorge forests and future changes in fog frequency over time are uncertain (Gutiérrez et al., 2008). Reductions in rainfall and fog inputs coupled to increasing patch fragmentation (Sala et al., 2000), will decrease the water budget of these forests because lower water capture surfaces and higher environmental variability. This scenario will expose *A. punctatum* trees to greater drought stress in this patch mosaic.

The inability of *A. punctatum* to modify xylem anatomy traits, associated with its problem to maintain leaf turgor in the face of decreasing SM at leeward edges (Salgado-Negret et al., 2013) could seriously impair the ability of *A. punctatum* to supply water to leaves for photosynthetic gas exchange. This mechanism could lead to a negative water balance and increased tree mortality along exposed patch edges and small size patches. Higher tree mortality would alter the hydrological balance of fragmented forests, affecting regeneration and persistence of other species that depend on ecosystem integrity. Global change, expressed in reductions of forest cover, increased fragmentation and more intense edge effects are likely to have strong negative impacts on forest ecosystems worldwide and on these fog-dependent ecosystems in particular, because of ecophysiological limitations and drought effects on the performance and survival of the dominant tree species.

## Author Contributions

BS-N, JA, and FP conceived and designed the research. BS-N and RC conducted fieldwork. BS-N, FV, FP analyzed the data. BS, RC, FV, JA and FP wrote the manuscript and approved the final version.

## Conflict of Interest Statement

The authors declare that the research was conducted in the absence of any commercial or financial relationships that could be construed as a potential conflict of interest.
